# Hyaluronic Acid Injection Laryngoplasty for Unilateral Vocal Fold Paralysis—A Systematic Review and Meta-Analysis

**DOI:** 10.3390/cells9112417

**Published:** 2020-11-05

**Authors:** Chen-Chi Wang, Shang-Heng Wu, Yu-Kang Tu, Wen-Jiun Lin, Shih-An Liu

**Affiliations:** 1 School of Medicine, National Yang-Ming University, Taipei 11221, Taiwan; 2 Department of Speech Language Pathology & Audiology, Chung Shan Medical University, Taichung 40201, Taiwan; 3 Department of Audiology and Speech-Language Pathology, Asia University, Taichung 41354, Taiwan; 4 Department of Otolaryngology-Head & Neck Surgery, Taichung Veterans General Hospital, Taichung 40705, Taiwan; entwsh@gmail.com (S.-H.W.); kgjack2001@gmail.com (W.-J.L.); saliu@vghtc.gov.tw (S.-A.L.); 5 Institute of Epidemiology and Preventive Medicine, College of Public Health, National Taiwan University, Taipei 100, Taiwan; yukangtu@ntu.edu.tw

**Keywords:** hyaluronic acid, injection laryngoplasty, unilateral vocal fold paralysis, meta-analysis

## Abstract

Unilateral vocal fold paralysis (UVFP) is a common disorder that may cause glottal closure insufficiency and then hoarseness of voice and aspiration during swallowing. We conducted a systematic review and meta-analysis to evaluate whether hyaluronic acid (HA) injection laryngoplasty (IL) is an effective treatment for patients with UVFP. Comprehensive systematic searches were undertaken using PubMed, EBSCO Medline, and Cochrane Library databases. We appraised the quality of studies according to preset inclusion and exclusion criteria. The lengths of follow-up were divided into “short-term” (3 months or shorter), “medium-term” (6 months), and “long-term” (12 months or longer). We performed random-effect meta-analysis to estimate the changes in voice-related quality of life, perceptual evaluation by grading systems, voice lab analysis of maximal phonation time, and normalized glottal gap area, before and after HA IL. Fourteen studies were eligible for the final analysis. The results showed that patients’ glottal closure insufficiency could be improved; maximal phonation time could be prolonged; perceptual evaluations of the voice and quality of life were better after HA IL, but the duration of treatment effect varied among different studies. In conclusion, HA IL is an effective treatment for UVFP, which may achieve a long-term effect and therefore reduce the likelihood of requiring permanent medialization thyroplasty.

## 1. Introduction 

Unilateral vocal fold paralysis (UVFP) due to recurrent laryngeal nerve (RLN) injury is a common disorder that may cause incomplete vocal fold adduction with glottal closure insufficiency, leading to hoarseness of the voice and aspiration during swallowing. This causes severe impairment of the patient’s quality of life. Apart from the use of voice therapy as a conservative form of management, surgical treatments are usually applied to improve glottal closure and to synchronize the vocal fold vibration during phonation. Although laryngeal reinnervation may increase the muscle tone of laryngeal muscles and the long-term maintenance of the vocal fold resistance necessary for glottis vibration during phonation, the effect of axonal growth occurs between months 4 and 6 after the reinnervation procedure and the beneficial effect on voice outcome takes a while to manifest. Therefore, it is recommended as a salvage option in UVFP [1]. Other surgical procedures, such as open laryngeal framework surgery and injection laryngoplasty (IL), are aimed at moving the paralyzed vocal fold closer to the glottis midline, enabling glottal closure during phonation and prompt restoration of vocal function after surgery. 

Open laryngeal framework surgery is a transcutaneous invasive surgery involving the creation of a window in the thyroid cartilage and the insertion of an implant to push the paralyzed vocal fold medially (medialization thyroplasty or thyroplasty type I) and/or adjust the position of arytenoid cartilage (arytenoid adduction) to improve glottal closure. Because RLN function may recover to some degree over the next several months after UVFP, many surgeons defer invasive open laryngeal framework surgery in the early stage of UVFP [2]. 

Compared to open laryngeal framework surgery, IL is relatively noninvasive without a cervical open approach and is usually done early after the onset of UVFP at a very low risk to the patient [2]. This procedure was first described by Brunings in 1911 [3], and there has been a continuous evolution in injection materials and injection techniques [4]. Xenograft, homograft, autograft, and synthetic materials, such as collagen, hyaluronic acid (HA), dermalogen, autologous fat, teflon, calcium hydroxylapatite, have been used as injection materials [5]. HA is a universal component of the extracellular matrix and was first introduced by Hertegard et al. for IL in glottal insufficiency caused by different etiologies including UVFP in 2002 [6]. It has a simple molecular structure with no structural differences across species from human to bacteria, and the lack of amino acids renders it non-immunogenic [7]. In addition, the vocal fold lamina propria compartment is a pauci-cellular layer that mostly contains the extracellular matrix and HA. The viscoelastic properties of vocal folds after the injection of HA-based material are similar to the healthy vocal fold in animal studies [8]. Owing to the aforementioned safety advantages, HA has become a commonly used material for IL. However, there are several different brands of HA fillers [9] and there are different molecular sizes [10].The injection techniques, outcomes, and complications related to HA also vary in the literature [4]. 

The aim of this systematic review was to synthesize evidence on the use of IL with HA for the treatment of UVFP. Meta-analyses of clinical outcomes were also undertaken to estimate the treatment effects of IL with different HA materials and injection techniques. Evidence on the safety of IL is also reviewed herein, and the results may be of value in clinical practice.

## 2. Materials and Methods

### 2.1. Protocol and Registration

Prior to undertaking the systematic review, we developed a protocol which was registered in the international prospective register of systematic reviews PROSPERO (https://www.crd.york.ac.uk/prospero/) [11], registration ID number 178226,entitled, “Hyaluronic acid vocal fold injection for patients with unilateral vocal fold paralysis-a systematic review”. The protocol was based on Preferred Reporting Items for Systematic Reviews and Meta-Analyses (PRISMA) guidelines [12]. 

### 2.2. Eligibility Criteria

We included original research studies that investigated the effect of HA IL for UVFP. We excluded case reports, review articles, and correspondence. The detailed inclusion and exclusion criteria are listed in Table 1.

### 2.3. Search Strategy

We conducted a comprehensive search of three databases, PubMed, EBSCO Medline, and Cochrane Library, without language restriction, from December 2002 (first published paper of Hertegard et al. [6]) to 5 April 2020. The following Medical Subject Headings (MeSH) terms and keywords were applied: (“hyaluronic acid” OR “hyaluronan”) AND (“injection”) AND (“unilateral vocal fold paralysis” OR “unilateral vocal fold palsy” OR “unilateral vocal cord paralysis” OR “unilateral vocal cord palsy”). Screening of studies by titles and abstracts was then undertaken. Duplicates were removed. Finally, full-text screening and evaluation was performed independently by two reviewers (Wang C.C. and Lin W.J.). If there were discrepancies, a third reviewer (Liu S.A.) then arbitrated. 

### 2.4. Data Extraction and Study Items

We developed a data extraction form to record the following key information: first author’s name, year of publication, country, study designs (retrospective, prospective, randomized control trial etc.), number of analyzed patients, anesthesia for injection, technique of injection, brand name of hyaluronic acid, injected volume, gauge of injection needle, pre- and post-injection evaluation, longest follow-up time after injection. Data extraction was independently performed by 2 authors (Wang C.C. and Lin W.J.), and any discrepancy was resolved by consensus-based discussion with a third reviewer (Liu S.A.). 

### 2.5. Assessment of Reporting Quality and Risk of Bias

The clinical outcomes in our meta-analyses were the quantitative evaluation of glottal function undertaken before and after the injection, including quality of life assessment by questionnaires, perceptual evaluation by grading systems, voice lab analysis of various acoustic and aerodynamic parameters, and image or video evaluation of glottal closure. As the included studies reported outcomes measured at different time points after treatments, we therefore divided the follow-up lengths into “short-term” (3 months or shorter), “medium-term” (6 months), and “long-term” (12 months or longer) to determine the trends of treatment effects. As the number of studies within each group of follow-up lengths was small, we did not, therefore, undertake an assessment of publication bias.

### 2.6. Data Synthesis and Meta-Analysis

The effect size measure is the difference in the outcome between the baseline and follow-up. For studies that only reported the baseline and the follow-up values, we assumed their correlation, r, to be 0.5 when calculating the standard deviation for the difference ($s_{\Delta }$) by using the following formula:

(1) $$s_{\Delta }=\sqrt{s_{B}^{2}+s_{F}^{2}-r*s_{B}*s_{F}}$$

where s_B_ and s_F_ are the standard deviations of the baseline and follow-up values, respectively. Because the included studies used different questionnaires for the assessment of quality of life, we therefore calculated the standardized mean gain and its standard error [13]. For the other three outcomes (perceptual evaluation by grading systems, voice lab analysis of various acoustic and aerodynamic parameters, image or video evaluation of glottal closure), the mean difference and the associated 95% confidence intervals were calculated. Preliminary analyses showed the between-study heterogeneity was large, so we used the DerSimonian and Laird random effects model for all of our meta-analyses [14]. All the statistical analyses were undertaken using the statistical software Stata (version 16.1, StataCorp, 4905 Lakeway Drive, College Station, TX, USA).

## 3. Results

### 3.1. Study Selection

**Figure 1:**
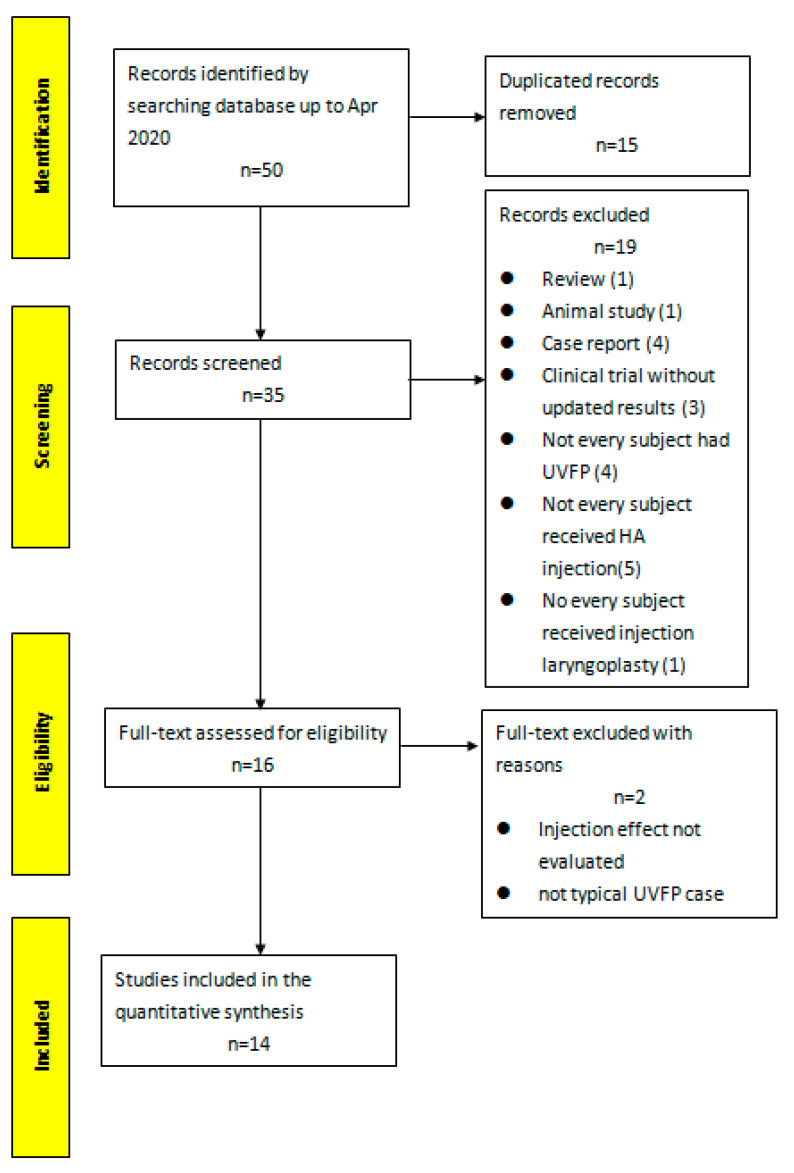
Flowchart diagram of literature search and selection. Inclusion and exclusion criteria are listed in Table 1.

In total, our literature search yielded 50 records, of which 15 were duplicates, resulting in 35 articles for further screening. After screening their titles and abstracts, 19 papers were excluded. Full texts of the remaining 16 articles were further evaluated and 14 articles were included in this systematic review and meta-analysis. The flow chart of the study selection process is shown in Figure 1. 

### 3.2. Study Characteristics

The included papers [15,16,17,18,19,20,21,22,23,24,25,26,27,28] were published between the years 2010 and 2018, and their characteristics are summarized in Table 2. As shown in Table 2, six studies were conducted prospectively, four retrospectively, and four without specific mention of their study design. The number of patients included in the meta-analysis ranged from 5 to 68. Regarding the anesthesia for IL, local anesthesia was used in nine (64.3%) studies, general anesthesia was used in three (21.4%) studies, anesthesia was spared in two (14.3%) studies. To guide the IL, a flexible laryngoscope was used in six (42.9%) studies, direct rigid laryngoscope was used in two (14.3%) studies, laryngeal electromyography (LEMG) was used in three (21.4%) studies, and information was unavailable for three studies. The injection approach could be divided into the trans-oral approach and trans-cutaneous cervical approaches. Trans-cutaneous cervical approaches were reported in nine (64.2%) studies, while the trans-oral approach was reported in two (14.3%) studies. One study reported using two approaches and two papers had no specific record. For the 10 papers that reported using trans-cutaneous cervical approaches, cervical injection was performed via crico-thyroid membrane in seven (70%) studies, via the thyro-hyoid membrane in one (7.1%) study, via the crico-thyroid or the thyro-hyoid membrane in one (7.1%) study, and the injection route was not reported in one study. For the trans-cutaneous cervical approach, injection via the crico-thyroid membrane could spare the vocal fold puncture by submucosal delivery of HA in seven (50%) papers. However, trans-cutaneous thyro-hyoid membrane injection and the trans-oral approach punctured the vocal fold membrane (trans-mucosa) in three (21.4%) papers.

Various HAs were used in the included studies. Restylane (Q-Med, Uppsala, Sweden) was used in seven (50%) studies; Restylane Perlane (Q-Med, Uppsala, Sweden) was used in four (30.8%) studies; the effect of Restylane and Restylane Perlane injection was compared in a study. Hyaluronic acid-dextranomer was used in one (7.1%) study. The effect of Restylane Perlane and collagen was compared in another study. In two studies, it is unclear which type of HA was used. For the volume of injected HA: less than 1cc or 1cc of HA was injected in seven (50%) studies, 2cc was used in one (7.1%) study, while no detailed information was reported in the other six studies. The needle gauge ranged from 21 to 27. The follow-up evaluation was undertaken as early as 3 days and up to 17.4 months after injection. For our meta-analyses, the longest follow-up length was divided into short-term (≤3 months) in four (28.5%) studies, medium-term (6 months) in four (28.5%) studies; and long-term (≥12 months) in six (42.9%) studies.

### 3.3. Pre-Injection and Post-Injection Glottal Function Evaluations

The evaluation tools used in different studies are summarized in Table 3. The evaluations included four main categories: quality of life questionnaires, perceptual evaluation grading system, voice laboratory recording, and image or video analysis. 

#### 3.3.1. Quality of Life Questionnaires

**Figure 2:**
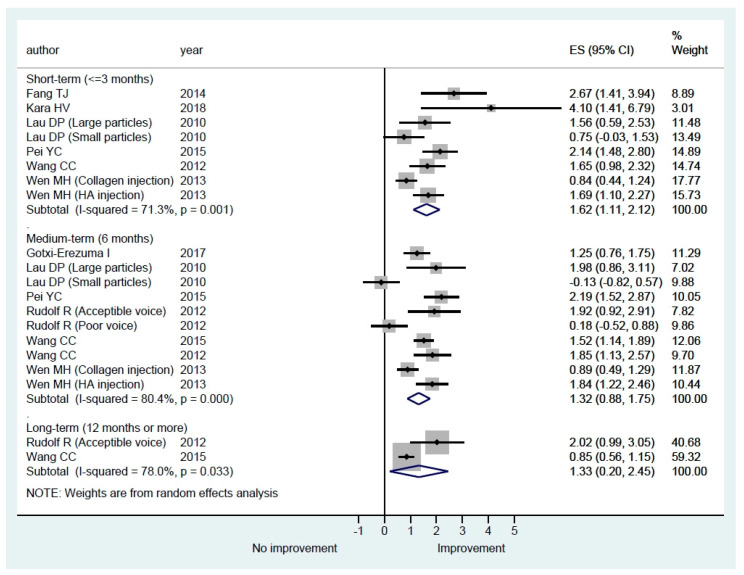
Subgroup meta-analysis of overall quality of life in short-term, medium-term, and long-term follow-up after hyaluronic acid injection laryngoplasty.

Voice handicap index (VHI) [29] was used in six (42.9%) studies (Table 3). The short version of VHI with 10 questions (VHI-10) [30] was used in two (14.3%) studies. Voice outcome survey (VOS) [31] was used in three studies. A decrease in VHI and VHI-10 is considered an improvement, whereas higher scores are better in VOS. We therefore reversed the sign of the change in VHI and VHI-10 before pooling the data for meta-analysis. Because the included studies used different questionnaires for the assessment of quality of life, we calculated the standardized mean gain and its standard error as the effect size. The results of our meta-analysis are shown in Figure 2. The small particles subgroup in the study by Lau [27] observed small benefits in the short term but not in the medium term follow-up and the poor voice subgroup in the study by Rudolf [25] observed no benefits in the medium-term follow-up. Except for these two groups, all of the other studies showed an improvement in quality of life in different follow-up periods. The pooled estimates of standardized mean gain were statistically significant in all three lengths of follow-up, but the between-study heterogeneity was large (Figure 2).

#### 3.3.2. Perceptual Evaluation Grading Systems

**Figure 3:**
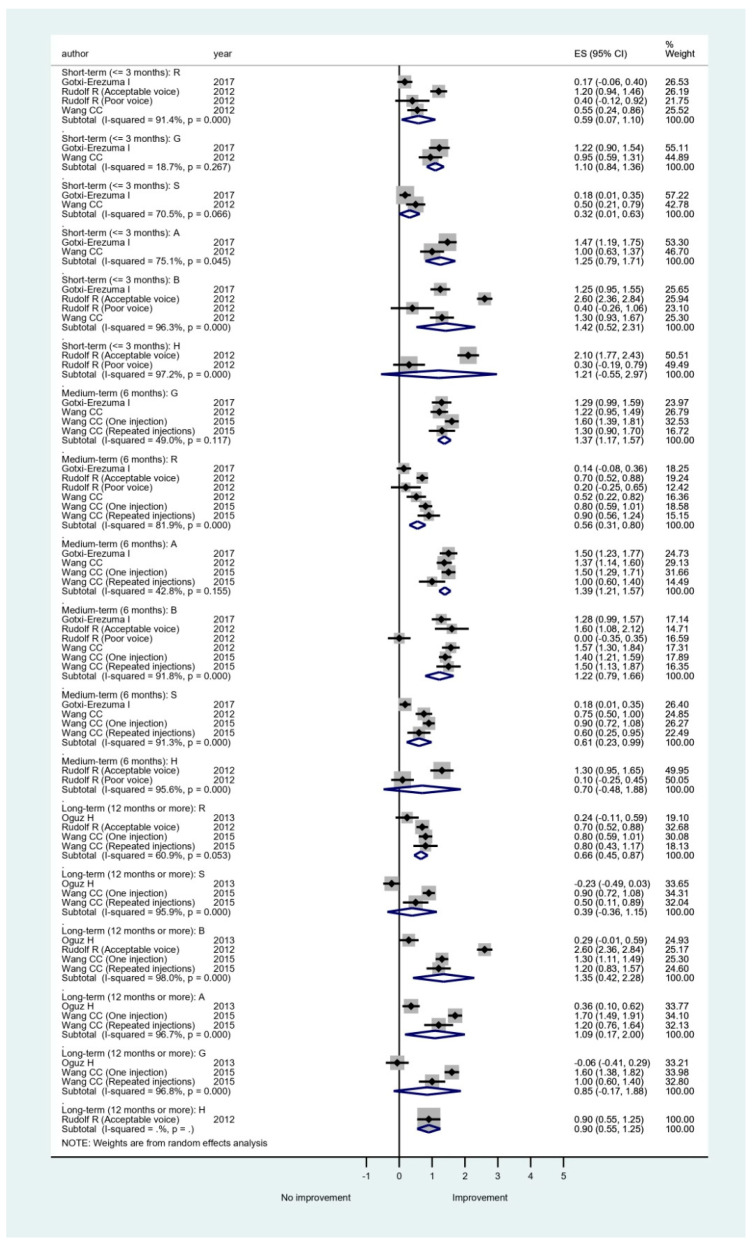
Subgroup meta-analysis of overall grading of different items of perceptual evaluation (G, R, B, A, S and H) in short-term, medium-term, and long-term follow-up after hyaluronic acid injection laryngoplasty.

The perceptual evaluation grading system of GRBAS, developed by the Japanese Society of Logopedics and Phoniatrics, was used in five (26.3%) studies (Table 3). GRBAS consists of five items (grade, roughness, breathiness, asthenia, strain) and uses the following scale for evaluation: 0(normal), 1(mild), 2(moderate), and 3(severe) [32]. Rudolf [25] used the RBH scale grading system, consisting of items for roughness, breathiness, and hoarseness, using the same 0–3 scale. We therefore conducted a meta-analysis for the items of GRBAS and H, and the results are shown in Figure 3. In the short-term, except H, the pooled estimates of mean differences were statistically significant in G, R, B, A, and S, suggesting an improvement in the perceptual evaluation. The medium-term results were similar to the short-term results. The long-term data showed that the pooled estimates of mean differences were statistically significant in R, B and A, but not in G, and S. There were no long-term data available for H. The between-study heterogeneity ranged from small (I^2^ = 18.7%) to large (I^2^ = 98%) in different subgroups.

#### 3.3.3. Voice Laboratory Recording

**Figure 4:**
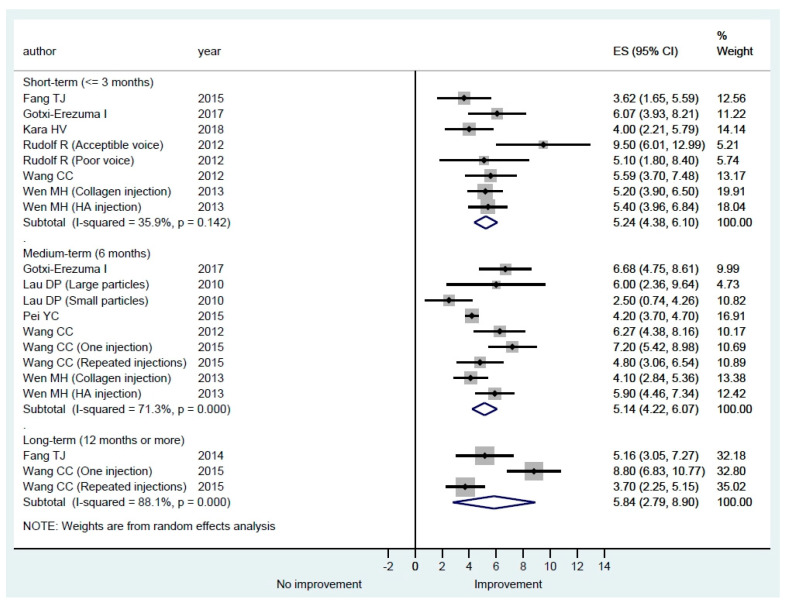
Subgroup meta-analysis of overall maximal phonation time (MPT) in short-term, medium-term, and long-term follow-up after hyaluronic acid injection laryngoplasty.

Numerous acoustic analyses can be conducted, including the collection of aerodynamic data, in a voice laboratory to objectively evaluate the quality of voice from different perspectives. In our systematic review, we noted that various parameters, such as pitch, fundamental frequency (F0), intensity, jitter (perturbation of frequency), shimmer (perturbation of intensity), pitch range, intensity range, mean air flow rate (MAFR), phonation quotient, ratio of phonation time in speaking S and Z (S/Z), harmonic to noise ratio (H/N), voice range profile (VRP), maximal phonation time (MPT) have been reported in different studies. The normal range of these parameters may vary among patients of different genders, ages, and races [33,34,35,36]. MPT in phonating vowel /a/ or /i/ is a relatively simple and reliable measurement [37] and was most commonly reported by the included studies (11 [78.5%]), while the other parameters were reported by only a few studies (Table 3). Therefore, only MPT data were pooled for meta-analysis and the results are shown in Figure 4. All of the studies showed an improvement in MPT in different follow-up periods. The pooled estimates of mean differences were statistically significant in all three lengths of follow-up, but the between-study heterogeneity was small in the short-term follow-up and large in the medium- and long-term follow-up (Figure 4).

#### 3.3.4. Image or Video Analysis

**Figure 5:**
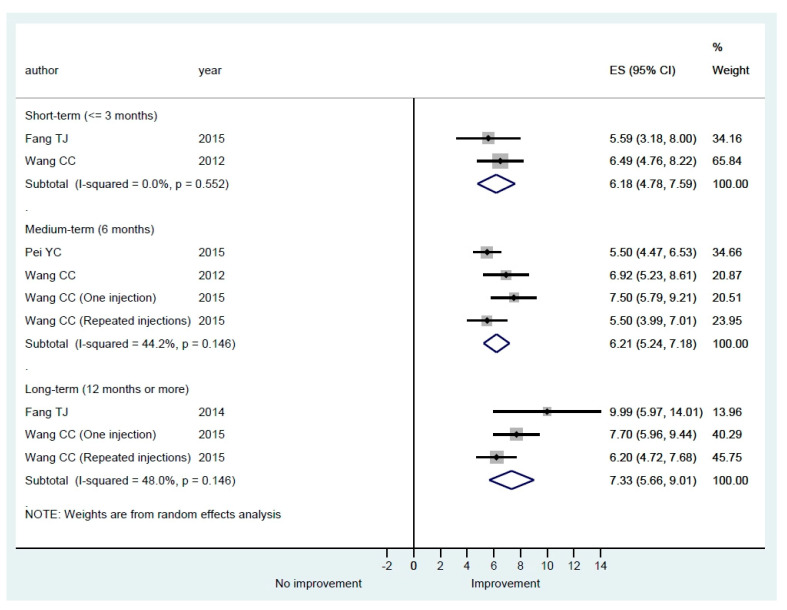
Subgroup meta-analysis regarding overall normalized glottal gap area (NGGA) in short-term, medium-term, and long-term follow-up after hyaluronic acid injection laryngoplasty.

UVFP may cause incomplete vocal fold adduction with glottal closure insufficiency. Therefore, the glottal gap during phonation is commonly measured to evaluate the severity of glottal closure insufficiency. In this systematic review, the normalized glottal gap area (NGGA), based on the definition by Omori et al. [38], was used in six (42.8%) studies to quantify the glottal gap. It was defined as the glottal gap area (pixels × pixels)/unaffected side membrane vocal fold length^2^ (pixels × pixels) × 100. Only one study, conducted by Lau et al., employed other measurements, such as glottic closed phase and glottis open fraction [27]. We therefore pooled the data of NGGA for meta-analysis and the results are shown in Figure 5. All of the included studies showed a reduction in NGGA and an improvement in glottal closure insufficiency. The pooled estimates of mean differences were statistically significant in all three lengths of follow-up, while the between-study heterogeneity ranged from small to moderate (Figure 5).

#### 3.3.5. Additional Evaluations

In addition to the aforementioned evaluations that were used to measure the change before and after IL with HA, other evaluations were done in some of the studies included in this systematic analysis. For example, in the study by Ng [15], a subtle improvement in the tone production of six Cantonese tones was found after the injection. The mean accuracy percentages in tone production before IL and 1 week, 1 month, and 3 months after IL were 59.43%, 69.91%, 63.27%, and 63.64%, respectively, compared with 81.55% in normal participants. In the study by Pei [16], laryngeal electromyography (LEMG) was used to measure the peak turn frequency of the thyroarytenoid–lateral cricoarytenoid muscle complex of paralyzed vocal fold. The voice range profile was also measured before and after IL. The peak turn frequency of the paralyzed thyroarytenoid–lateral cricoarytenoid muscle complex showed a modest correlation with maximal fundamental frequency and fundamental frequency range before injection. Therefore, it was concluded that change in voice pitch in patients with UVFP can partly predict impairment of neuromuscular functions. The study also found that IL with HA improved voice range profile performance by increasing the maximal fundamental frequency, decreasing the minimal fundamental frequency, and increasing fundamental frequency range. In the studies of Gotxi-Erezuma and Wang [18,21,26], LEMG was used for guiding HA injection and predicting the prognosis. The information obtained from LEMG could be used to help decide between repeated injection for patients with recovery potential or medialization thyroplasty for patients with poor prognosis if HA absorption occurred in the future. 

In another study by Pei [20], the HA injection group was compared with a conservative management group. At the end of a 6-month follow-up, improvements were found in most quality of life domains and other assessments were comparable between two groups, but the injection group had a greater improvement in the mental health quality of life domain. In a study by Fang [22], the conservative management group with NGGA > 7.36 had a higher rate of subsequent permanent medialization thyroplasty, but early HA injection may reduce the need of permanent medialization thyroplasty in patients with a large NGGA > 7.36. In a study by Freidman [28], the effect of early injection within 6 months of symptoms onset appeared to be better than those achieved with late injection. In the three patients who underwent late injection (>6 months post-paralysis), none avoided open surgery, while 20 of 32 patients (62.5%) with early injection (<6 months post-paralysis) maintained an adequate voice without the need of open surgery. In a study by Wen [23], a group of patients received IL with porcine collagen. They found comparable subjective and objective improvement compared to the results of patients receiving HA (Restylane Perlane) injection. In a study by Lau [27], compared to small particle size HA injection, large particle size HA injection tended to result in lower Voice Handicap Index scores (better voice), suggesting that large particle-size HA may be more appropriate for IL. 

The relationships between outcomes and other clinical variables, such as sex, age, type of anesthesia, transcervical versus transoral approaches, and so on, were analyzed. The results showed that the proportion of males in any given study was not correlated with the mean MPT in that study (*p* = 0.746). The mean age in studies was negatively associated with the mean MPT (−0.20 per year older, 95% CI: −0.39 to −0.02; *p* = 0.033). The proportion of males in a study was not correlated with the mean QOL (*p* = 0.469), nor was the mean age across studies (0.807). The proportion of males in a study was not correlated with the mean NGGA in a study (*p* = 0.598), nor was the mean age in studies (0.481). The mean MPT in studies with local anesthesia/no anesthesia was 0.69 s (95% CI: −5.48 to 4.10; *p* = 0.708) shorter than that in studies with general anesthesia, but the difference was not significant. The mean MPT in studies of the transcervical approach was 1.07 s (95% CI: −7.19 to 5.06; *p* = 0.532) shorter than that in studies of the transoral approach, but the difference was still not significant. However, as the number of studies was small, the statistical power of the meta-regression was low. 

## 4. Discussion

UVFP potentially leads to considerable morbidity including dysphonia, dysphagia, and aspiration. According to a systematic review conducted by Siu [39], four surgical interventions including IL, medialization thyroplasty, arytenoid adduction, and laryngeal reinnervation are available for the treatment of UVFP. Although each treatment has its pros and cons, there were no significant differences in outcomes among these treatments. In addition, IL is the only procedure which can spare an open approach. A systematic review and meta-analysis by Vila [2] suggested that early injection laryngoplasty (<6 months post-paralysis) may lower the rate of subsequent medialization thyroplasty. Therefore, IL has become an increasingly popular modality for promptly rehabilitating laryngeal function after onset of UVFP. 

In the previous literature, many injection materials have been used for IL. Kwon [5] indicated that an ideal IL material should be biocompatible, biomechanically similar to vocal fold components, easily injectable through a fine needle, readily available with minimal preparation time, applicable in an outpatient setting, easily removable in the event of revision surgery, and resistant to absorption or migration. Vocal fold injection with materials containing HA have been reported in animal studies with promising outcomes since 1998 [40]. With the exception of resistance to absorption and migration, HA seems to meet the aforementioned criteria for an ideal IL material. In 2002, Hertegard [6] started using cross-linked HA for treating glottal closure insufficiency caused by UVFP and vocal fold atrophy. They found the patients’ self-ratings of voice were significantly improved at 12 months after injection of HA and collagen. However, patients receiving HA injection showed better vocal fold status and longer maximal phonation time. Unfortunately, in the review by Lakhani [41], the authors found no high quality randomized controlled trials on the outcomes of different injection materials for UVFP. In this study, we restricted the focus of our review to HA IL and performed a systematic analysis of its safety, injection techniques, and different evaluation outcomes in different follow-up periods for UVFP. 

### 4.1. Safety and Complications of HA IL 

HA is a polysaccharide and is a universal component of the extracellular matrix. It is not species specific and does not elicit any humoral or cell-mediated immune response. There are two commercially available products: Hylan B gel (Hylaform, Biomatrix, Ridgefield, NJ, USA) and Restylane (Q-Med, Uppsala, Sweden) commonly used for IL [6,15,16,18,19,20,21,22,23,25,26,27]. Hyaluronan dextranomer (Deflux^®^), which is commonly used for treating pediatric vesicoureteral reflux has also been used for IL. [24,40,42]. Generally speaking, HA is quite safe for IL and only one patient experienced hematoma [18] and one patient experienced edema of the aryteno-epiglottic fold and false vocal fold [25] in our review of 14 studies. However, Hamdan [43] and Dominguez [44] described complication rates of 4.7% and 3.8% in their studies of IL for different indications including UVFP. The most common adverse reactions are local hypersensitivity and inflammation. The hypothesized mechanisms for adverse reaction include (1) local hypersensitivity to the proteins incidentally produced in the HA manufacturing process, (2) vascular compression or occlusion by the injected HA, and (3) contamination of the injector device. Although, to date, no deaths have been reported following treatment with IL, adverse reactions may induce local edema, erythema, induration, tenderness, abscess formation, and decreased vocal fold pliability, among others, leading to dysphonia, dyspnea, or dysphagia. Although adverse reactions may be treated with steroids or antibiotics, the length of adverse reaction may still range from hours to days, weeks to months, or may even have long-term sequelae without resolution (26 months) [44]. 

### 4.2. Techniques of HA IL

**Figure 6:**
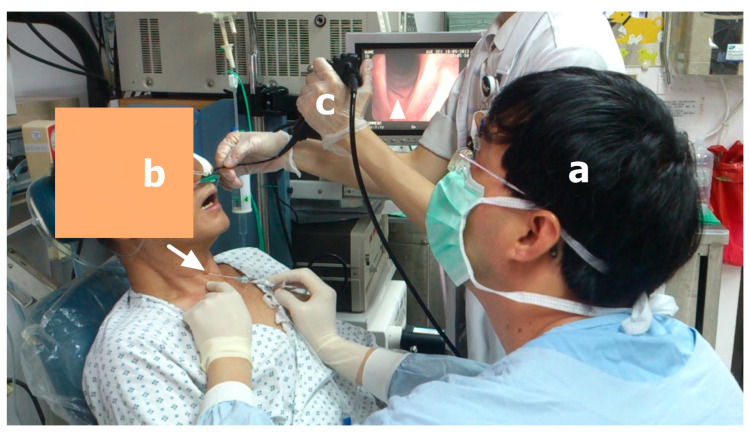
Hyaluronic acid injection laryngoplasty (arrow head) under local anesthesia, guided by flexible laryngoscopy, using the trans-cervical approach via cricothyroid membrane puncture (arrow). (**a**) Surgeon; (**b**) a patient in sitting position; (**c**) an assistant controls the scope.

**Figure 7:**
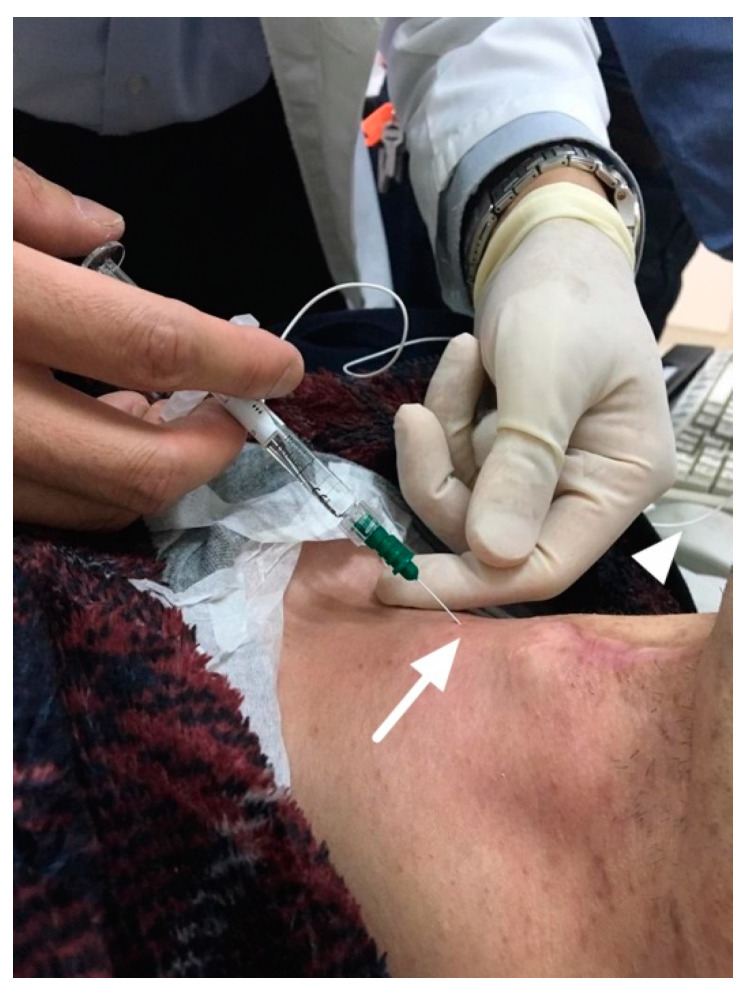
Hyaluronic acid injection laryngoplasty without anesthesia, guided by laryngeal electromyography injectable needle electrode connected to electromyography machine (arrow head). The injection was also done by the trans-cervical approach via cricothyroid membrane (arrow).

According to Table 2, for IL under local anesthesia, guided by flexible laryngoscopy, the trans-cervical approach via cricothyroid membrane puncture is the most commonly used technique in current studies (Figure 6). The aforementioned results are consistent with the findings of the study by Sulica [4]. This technique provides many clinical advantages. For example, it provides the shortest distance to deliver HA into the vocal fold and therefore surgeons can use a short, thin needle to perform the injection with minimal invasiveness. In addition, this technique avoids the risk of vocal fold trans-mucosa puncture and reduces the cough and gag reflex induced by mucosa irritation. However, flexible laryngoscopy guidance still needs local anesthesia and Sulica [4] noted that it is possible to anesthetize excessively, leading to salivary secretions that may overwhelm the larynx, making the patient uncomfortable and obscuring the injection site. In their study, patient discomfort accounted for the largest group of awake injection failure. In addition, this practice requires an assistant capable of skillfully controlling the scope and working in close cooperation with the surgeons. Furthermore, it is clear that, even under laryngoscopy guidance, the submucosal passage of the needle still cannot be seen directly. To overcome the disadvantages of scope guidance, IL guided by LEMG was first developed by Wang [26] in 2012 (Figure 7). This technique simplifies IL by obviating the need for a scope, a laryngoscopy assistant, and even local anesthesia. It provides an alternative way to maintain the needle tip in the thyro-arytenoid and lateral circo-arytenoid muscle complex of vocal fold and to finish the injection for patients in a comfortable supine position. The feasibility of this approach was also confirmed in a study by Gotxi-Erezuma [18]. However, many laryngologists do not routinely use LEMG, even though it has been considered a valuable diagnostic tool for more than 60 years [45]. Therefore, Wang [46] proposed LEMG-guided IL to enhance the popularity of LEMG. If surgeons perform IL for UVFP via an injectable needle electrode during LEMG, diagnosis, prognosis prediction, and treatment can be achieved in one step. They found that LEMG has a high positive predictive value in predicting the long-term outcome of UVFP patients with a poor prognosis [47]. If the effect of HA injection diminishes, the prognostic information obtained from LEMG can be used as guidance for future open laryngeal framework surgery [46]. Permanent medialization thyroplasty is feasible if patients have positive findings on LEMG at least 2 months after symptom onset. 

### 4.3. Different HA, Volume, and Needle

As mentioned above, three HA-based materials are available for IL. Hyaluronan dextranomer (Deflux^®^) is mainly used for vesicoureteral reflux. The detranomer microspheres have an average size of 130 microns. The stabilized HA acts mainly as a carrier, leaving the detranomeres at the implant site. Hylaform is a nonpyrogenic, clear, colorless gel implant created through the reaction of divinyl sulfone with hyaluronan hydroxyl groups (from rooster combs). Restylane is a non-animal, stabilized hyaluronic acid (NASHA), which is biotechnologically generated by Streptococcus species of bacteria and chemically cross-linked with diepoxide 1, 4-butanediol diglycidyl ether. Both were developed as dermal filler, but a study by Caton [48] showed that Hylaform and Restylane share comparable elastic values and low viscosity that are similar to a cadaveric vocal fold. Hylaform and Restylane have family products of different particles sizes. For example, Hylaform had a smaller particle size similar to that of Restylane. Hylaform plus and Restylane Perlane are cross-linked HA with larger particle sizes. The results from a study by Lau [27] show that large particle size HA is more durable than small particle HA for IL. However, HA of different particle sizes could be injected through a thin needle ranging from 21 G to 27 G in this study. Regarding the injected volume of HA required to obtain satisfactory results, the findings of our review showed that the injected volume was usually less than or equal to 1 mL. Only Gotxi-Erezuma [18] used 2 mL of Restylane Perlane for injection. It seems that 1 mL of HA was usually enough to achieve overcorrection of a paralyzed vocal fold and the shape of an over-injected vocal fold remodels in response to the compression from the contra-lateral mobile vocal fold [26]. 

### 4.4. Treatment Outcomes 

Theoretically, any management that could reduce the glottal gap of UVFP could potentially improve the patient’s voice and quality of life. Normalized glottal gap area or NGGA is a standardized method that directly evaluates the glottal gap of UVFP. The pooled estimates of mean differences were statistically significant in all three lengths of follow-up in our reviews (Figure 5). The results confirmed that vocal fold augmentation is effective at improving glottal closure. In addition to improved glottal closure, our meta-analysis results also showed that HA IL may improve quality of life after injection (Figure 2). However, the between-study heterogeneity was large and thus the results need to be interpreted with caution. For perceptual evaluation in the short-term and medium-term, except H, the scale of G, R, B, A, and S improved significantly. Because absorption of HA will occur in some treated patients, some perceptual scales such as G and S may improve non-significantly in the long-term (Figure 3). In Figure 4, average MPT could increase by around 5 s in all lengths of follow-up. The between-study heterogeneity was not large, especially in the short-term. The overall results show that IL with HA is beneficial for UVFP patients mostly in the short-term and medium-term. There are studies showing long-term benefit in some patients after HA injection [21,22,23,24,25]. There are three possible mechanisms of the long-term effect obtained from injection laryngoplasty with an absorbable HA [21]. First, Restylane Perlane has been reported to undergo isovolemic degradation, which involves aggregation of more water as it is absorbed [49]. Second, laryngeal reinnervation of paralyzed adductor muscles and synkinesis may account for a medial position of the paralyzed vocal fold. The atrophic thyroarytenoid muscle could also regain some muscle bulk after regeneration [50]. Third, during the gradual absorption of injected HA in some patients, the contralateral healthy vocal fold probably compensates for the glottis closure. However, the occurrence of the aforementioned three scenarios is unpredictable and HA injection laryngoplasty does not guarantee long-term improvement. 

### 4.5. Study Limitations 

A wide range of studies have been published on the topic of UVFP, but only a small number of studies investigated the use of HA IL. In real-world practice, there is no standard procedure of injection laryngoplasty for UVFP. We have observed substantial differences in the injection techniques between institutions and Sulica et al. have commented that the choice of technique is usually based on the surgeon’s preference [4]. In addition, there is no standardized outcome evaluation protocol. The interpretation of the pooled data was therefore challenging because of the considerable variations across the studies in their study design and methodology. The lack of standardization in outcome surveys means that many results are based on large between-study heterogeneity. Furthermore, there was no control group of patients without injection laryngoplasty in our reviewed studies. This may be explained by the fact that existing studies suggest early injection may be significantly associated with a lower rate of eventual need for permanent open surgery [22,28,51]. Moreover, patients tend to expect prompt improvement after the onset of UVFP symptoms, and minimal injection laryngoplasty was designed for early rehabilitation. Therefore, it remains a considerable challenge to investigate this topic using a meta-analysis of randomized controlled trials. This review is certainly limited by the quality of the included studies. We therefore conducted a comprehensive review of reported HA IL in the literature. We recommend that clinicians consider their clinical skills and available facilities when deciding on the best approach.

## 5. Conclusions

HA IL has been used for treating UVFP for more than 10 years. It can be done in a clinic using flexible laryngoscopy or LEMG guidance. Under local anesthesia, about 1 mL of commercially available HA can be delivered by a thin needle up to 27 G. The most common approach is trans-cervical trans-cutaneous puncture via the cricothyroid membrane to deliver HA into the submucosa region of the vocal fold. After treatment, patients’ glottal closure insufficiency can be improved and their maximal phonation time can be prolonged with increased voice range performance. Perceptual evaluations of the voice and patients’ quality of life can also be enhanced. The duration of treatment effect varied in different studies, and the long-term effects were observed in some patients. Early injection after symptom onset could reduce the rate of permanent medialization thyroplasty. A combination of LEMG guidance, diagnosis, treatment, and prognosis prediction can be obtained in one procedure. 

## Figures and Tables

**Table 1:** Inclusion and exclusion criteria that were applied in this systematic review.

Inclusion Criteria	Exclusion Criteria
Original research articles	The retrieved records were ⮚ Case reports ⮚ Reviews ⮚ Correspondence ⮚ Unpublished studies without available data
Human studies	Animal studies
Patients diagnosed with unilateral vocal fold paralysis (UVFP)	Patients with UVFP mixed with other etiologies of glottal closure insufficiency
Vocal fold injection material was hyaluronic acid	Injection material mixed with other materials
Quantitative evaluation of glottal function before and after injection	Without clear quantitative evaluation of glottal function before and after injection

**Table 2:** Summary of the characteristics of reported articles discussing hyaluronic acid injection laryngoplasty for unilateral vocal fold paralysis.

Number + References	Year	Nation	1st Author	Retrospective or Prospective	Patient Number	Anesthesia Type	Injection Guidance	Injection Approach	Skin Injection Route	Vocal Fold Injection	HA Name	Injected Volume	Gauge of Needle	Men F-U (Months)
1 [15]	2018	Hong kong	Ng ML	NA	30	Local	Flexible L-S	Trans-cutaneous	NA	NA	Restylane	NA	NA	3
2 [16]	2018	Taiwan	Pei YC	Prospective	68	Local	Flexible L-S	Trans-cutaeous	CT	Sub-mucosa	Restylane	1cc	NA	1
3 [17]	2018	Turkey	Kara HV	Retrospective	5	General	Direct L-S	Trans-oral	nil	Trans-mucosa	NA	1cc	25	3 days
4 [18]	2017	Spain	Gotxi-Erezuma I	NA	28	Local	LEMG	Trans-cutaneous	CT	NA	Perlane	2cc	23	6
5 [19]	2015	Taiwan	Fang TJ	Retrospective	34	Local	Flexible L-S	Trans-cutaeous	CT	Sub-mucosa	Restylane	1cc	NA	1
6 [20]	2015	Taiwan	Pei YC	NA	29	Local	Flexible L-S	Trans-cutaeous	CT	Sub-mucosa	Restylane	NA	23 or 25	6
7 [21]	2015	Taiwan	Wang CC	Prospective	60	Spared	LEMG	Trans-cutaeous	CT	Sub-mucosa	Perlane	1cc	26	17.4
8 [22]	2014	Taiwan	Fang TJ	Prospective	20	Local	Flexible L-S	Trans-cutaeous	CT	Sub-mucosa	Restylane	NA	NA	15
9 [23]	2013	Taiwan	Wen MH	NA	60	Local	NA	Both	TH	Trans-mucosa	Perlane (27) + collagen (33)	0.5 to 1cc	NA	15
10 [24]	2013	Turkey	Oguz H	Retrospective	17	General	Direct L-S	NA	nil	NA	Hyaluronan dextranomere	NA	NA	14
11 [25]	2012	Germany	Rudolf R	Prospective	19	General	NA	NA	NA	NA	Restylane	0.9 cc	NA	12.6
12 [26]	2012	Taiwan	Wang CC	Prospective	20	Spared	LEMG	Trans-cutaeous	CT	Sub-mucosa	Perlane	1cc	26	6
13 [27]	2010	Singapore	Lau DP	Prospective	17	Local	Flexible L-S	Trans-cutaeous	CT or TH	Sub-mucosa	Restylane (8) + Perlane (9)	NA	21 or 27	6
14 [28]	2010	USA	Friedman AD	Retrospective	35	Local	NA	Trans-oral	nil	Trans-mucosa	NA	NA	NA	15

CT: cricothyroid membrane; F-U: follow-up; LEMG: laryngeal electromyography; L-S: laryngoscope; NA: not available; TH: thyrohyoid membrane.

**Table 3:** Summary of the evaluations before and after hyaluronic acid injection laryngoplasty for unilateral vocal fold paralysis in reported articles.

Number + References	1st Author	QoL	Perceptual	Voice Laboratory	Image or Video	Additional Evaluations
1 [15]	Ng ML	VHI	GRBAS	Pitch,	nil	Tone production
2 [16]	Pei YC	nil	nil	MPT, F0, Jitter, shimmer, H/N, S/Z, VRP	NGGA	Q-LEMG
3 [17]	Kara HV	VHI-10	nil	MPT	nil	nil
4 [18]	Gotxi-Erezuma I	VHI	GRBAS	MPT	unknown	LEMG
5 [19]	Fang TJ	VOS; SF-36	nil	MPT, S/Z, F0, Jitter, shimmer, H/N	NGGA	Q-LEMG
6 [20]	Pei YC	VOS; SF-36	nil	MPT, S/Z, F0, Jitter, shimmer, H/N	NGGA	Compare conservation management, Q-LEMG;
7 [21]	Wang CC	VHI	GRBAS	MPT, PQ, MAFR	NGGA	LEMG
8 [22]	Fang TJ	VOS; SF-36	nil	MPT, S/Z, Jitter, shimmer	NGGA	Compare conservation management, Q-LEMG;
9 [23]	Wen MH	VHI-10	nil	MPT	nil	Compare HA and collagen injection
10 [24]	Oguz H	nil	GRBAS	F0, jitter, shimmer, HN	nil	nil
11 [25]	Rudolf R	VHI	RBH scale	MPT, intensity, F0, jitter, shimmer, VRP	nil	Compare acceptable voice + bad voice group
12 [26]	Wang CC	VHI	GRBAS	MPT, PQ, MAFR	NGGA	Choking scale
13 [27]	Lau DP	VHI	nil	MPT, MAFR, Jitter, shimmer, N/H, pitch range, intensity range	Glottic closed phase, Golttic open fraction	Compare large + small particle HA
14 [28]	Friedman	nil	nil	nil	nil	Compare early injection + late injection group

F0: fundamental frequency; H/N: harmonic to noise ratio; LEMG: laryngeal electromyography; MAFR: mean air flow rate; MPT: maximal phonation time; NGGA: normalized glottal gap area; PQ: phonation quotient; Q-LEMG: quantitative laryngeal electromyography; QoL: quality of life; S/Z: the ratio of the voicing duration of /s/ to /z/; VHI: voice handicap index; VOS: voice outcome survey; VRP: voice range profile.
